# Phenotypic findings and pregnancy outcomes of fetal rare autosomal aneuploidies detected using chromosomal microarray analysis

**DOI:** 10.1186/s40246-022-00438-4

**Published:** 2022-12-01

**Authors:** Rong Hu, Weiwei Huang, Weining Zhou, Xiaohui Luo, Congmian Ren, Huajie Huang, Yaping Hou, Li Guo, Wei He, Jian Lu

**Affiliations:** 1 Medical Genetic Center, Guangdong Women and Children Hospital, No.521, Xingnan Road, Panyu District, Guangzhou, 511400 Guangdong China; 2 Maternal and Children Metabolic-Genetic Key Laboratory, Guangdong Women and Children Hospital, Guangzhou, 511400 China

**Keywords:** Rare autosomal aneuploidy, Chromosomal microarray analysis, Chromosomal mosaicism, Prenatal diagnosis

## Abstract

**Background:**

Aneuploidies are the most common chromosomal abnormality and the main genetic cause of adverse pregnancy outcomes. Since numerous studies have focused on common trisomies, relatively little is known about the association between phenotypic findings and rare autosomal aneuploidies (RAAs). We conducted a retrospective study of 48,904 cases for chromosomal microarray analysis in a large tertiary referral center and reported the overall frequencies, clinical manifestations, and outcomes of prenatal RAAs.

**Results:**

A total of 90 RAAs were detected, of which 83 cases were mosaic trisomies and 7 were non-mosaic trisomies. Chromosomes 16, 22, and 9 were identified as the major chromosomes involving RAAs. The four predominant indications for prenatal diagnosis in our RAA cases were RAA-positive in noninvasive prenatal screening, advanced maternal age, ultrasound abnormalities, and high-risk for serum prenatal screening. Cardiovascular defects were the most frequently observed structural abnormalities, followed by musculoskeletal anomalies. Increased nuchal translucency and persistent left superior vena cava, the major soft marker abnormalities involved, were also observed in our RAA cases. Clinical outcomes were available for all RAAs, with 63 induced abortions and 27 live births recorded.

**Conclusions:**

Variable phenotypes and outcomes were observed, which were highly heterogeneous in cases of prenatal RAAs. Thus, a cautious and comprehensive strategy should be implemented during prenatal counseling for RAAs.

**Supplementary Information:**

The online version contains supplementary material available at 10.1186/s40246-022-00438-4.

## Background

Rare autosomal aneuploidies (RAAs), which involve autosomes other than 21, 18, and 13, are fairly uncommon during pregnancy and at birth. As RAAs are fatal, spontaneous abortion occurs most often during the first trimester [1]. Only a few rare mosaic trisomy embryos or fetuses can proceed to the second or third trimester [1, 2]. Hence, inadequate information is available regarding prenatal RAAs. In 1997, the associations between phenotypes and rare mosaic trisomies in amniocytes were studied [3]. Based on previous reports, Wallerstein et al. elucidated the phenotypes and outcomes of mosaic trisomies, providing an in-depth understanding of karyotype–phenotype correlations [4]. Although the majority of prenatal RAAs may have normal pregnancy outcomes [5, 6], RAAs remain a high-risk factor for abnormal complications, including miscarriage, confined placental mosaicism (CPM), intrauterine growth retardation (IUGR), and uniparental disomy (UPD) [7, 8]. Numerous published reports based on noninvasive prenatal screening (NIPS) using maternal plasma cell-free DNA (cfDNA) sequencing [9–11], the common screening method for RAAs, have described the frequency of RAAs and pregnancy outcomes, with results varying considerably. Moreover, the false-positive results of NIPS and the lack of confirmatory invasive testing make counseling on the phenotypic effects of RAAs more challenging. There are limited accurate data regarding whether prenatal RAAs are related to abnormal pregnancy outcomes or specific phenotypic findings. Therefore, the genotypic–phenotypic correlations of RAAs remain unclear.

With significant progress in prenatal diagnosis technologies in the past decades, cytogenetic and molecular genetic techniques, including karyotyping, fluorescence in situ hybridization (FISH), quantitative fluorescent polymerase chain reaction, chromosomal microarray analysis (CMA), and copy number variant sequencing, have been widely applied for the detection of chromosomal abnormalities [4, 5, 12–14]. As RAAs are most often mosaic in the second or third trimester, cytogenetics combined with molecular genetic diagnostic methods are commonly recommended in cases of suspected mosaicism [4]. CMA, a first-tier diagnostic method for fetuses with ultrasound-detected structural abnormalities [15], shows a high diagnostic performance sensitivity for prenatal mosaicism [16–18]. Accordingly, CMA plays a crucial role in the prenatal diagnosis of aneuploidies.

In this study, we comprehensively and systematically analyzed the data on RAAs detected by CMA over the past 4 years in the Guangdong Women and Children Hospital. The overall frequencies, clinical manifestations, and outcomes are described herein to provide a reference of prenatal RAAs for managing pregnancies.

## Results

### Demographics data

A total of 48,904 cases, including a mass of samples received from primary hospitals, were selected for invasive prenatal diagnostic detection via CMA at our center. Consequently, 90 cases (0.18%, 90/48,904) involving RAAs were detected in this study. In addition, 1,928 cases (3.94%, 1,928/48,904) of common aneuploidies were included, involving chromosome 21 (*n* = 1,336, 69.29%), chromosome 18 (*n* = 465, 24.12%), and chromosome 13 (*n* = 127, 6.59%). As given in Table 1, the median maternal age of patients with common aneuploidies was significantly higher than that of the RAA group (*p* < 0.05). Moreover, the RAA group had a higher ratio of mosaic trisomies than the common aneuploidy group (*p* < 0.001). In the RAA group, 71 cases (78.89%) were detected from AF, 17 (18.89%) from CVS, and 2 (2.22%) from CB. AF was the most frequent specimen type in this study.

**Table 1:** Demographics and specimen types in cases with common aneuploidies for chromosomes 21, 18, and 13 and RAAs

Characteristics	Common aneuploidies (*n* = 1928)	RAAs (*n* = 90)	*p* value
Maternal age (years)	34 (28–39)	32 (28–37)	*p* < 0.05
≥ 35 years old	954 (49.48%)	35 (38.89%)	0.052
Gestational ages (weeks)	18 (17–19)	18 (16–20)	0.568
Specimen types			
Chorionic villus	301 (15.61%)	17 (18.89%)	–
Amniotic fluid	1532 (79.46%)	71 (78.89%)	–
Cord blood	95 (4.93%)	2 (2.22%)	–
Classification			
Mosaic trisomies	69 (3.58%)	83 (92.22%)	*p* < 0.001

*p* value: comparisons were made between the common aneuploidies and RAAs group

### RAA detection via CMA

**Fig. 1:**
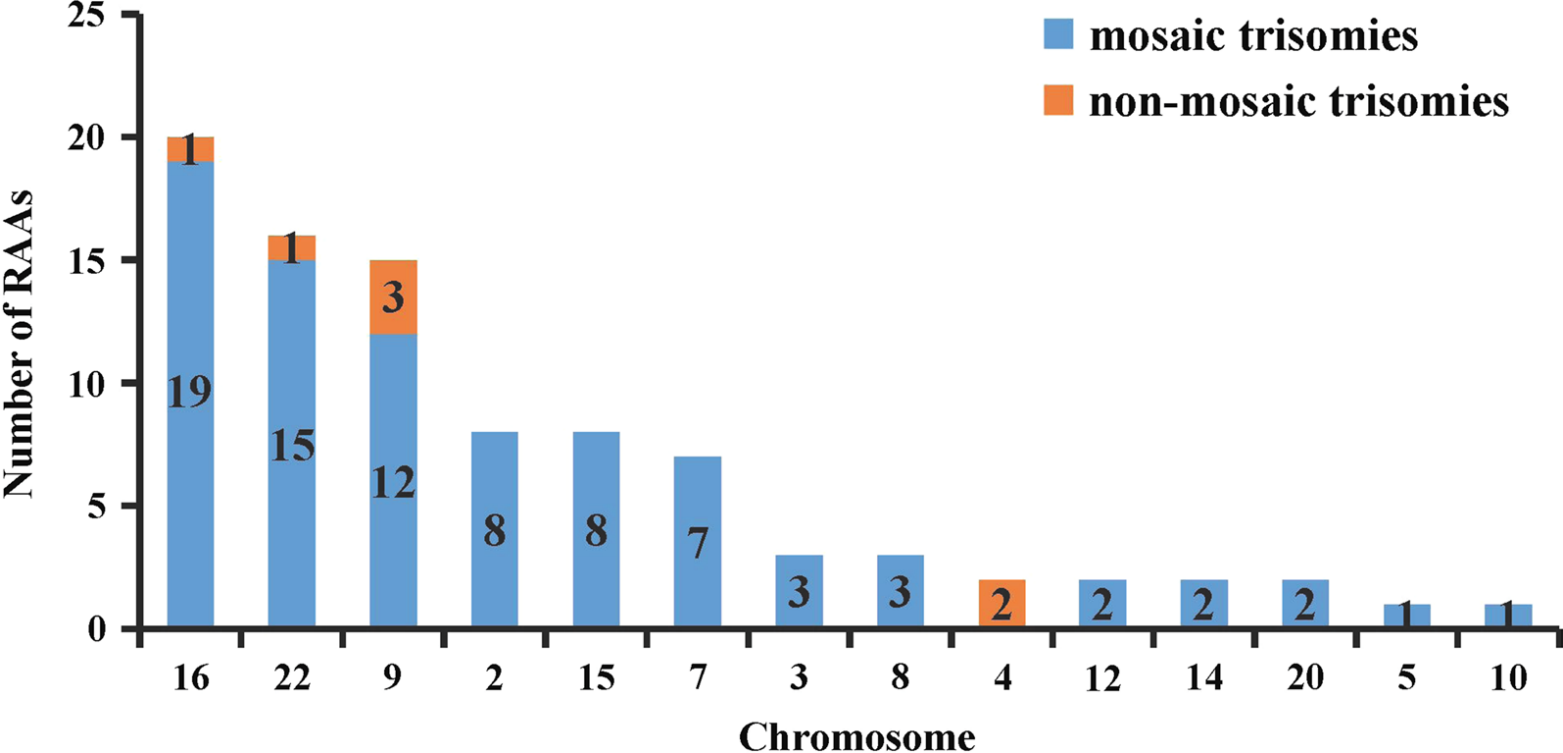
Chromosomal distribution of RAAs

In total, 90 RAAs were identified through CMA detection, of which 83 cases (92.22%) were mosaic trisomies and 7 (7.78%) were non-mosaic trisomies. A wide range of chromosomes were involved, including chromosomes 2, 3, 4, 5, 7, 8, 9, 10, 12, 14, 15, 16, 20, and 22. The chromosomal distribution of RAAs is shown in Fig. 1. The most common RAAs involved chromosome 16 (*n* = 20), followed by chromosome 22 (*n* = 16), chromosome 9 (*n* = 15), chromosome 2 and 15 (each *n* = 8), and chromosome 7 (*n* = 7). Less frequent RAAs involved chromosomes 3 and 8 (each *n* = 3); chromosomes 4, 12, 14, and 20 (each *n* = 2); and chromosomes 5 and 10 (each *n* = 1). Moreover, among the 90 RAAs, 7 were non-mosaic trisomies found in chromosome 9 (detected from CVS = 2 and AF = 1), chromosome 4 (detected from CVS = 2), chromosome 16 (detected from CVS = 1), and chromosome 22 (detected from CVS = 1). Notably, RAAs were not observed in chromosomes 1, 6, 11, 17, and 19 in this study.

### Clinical manifestations and outcomes

**Fig. 2:**
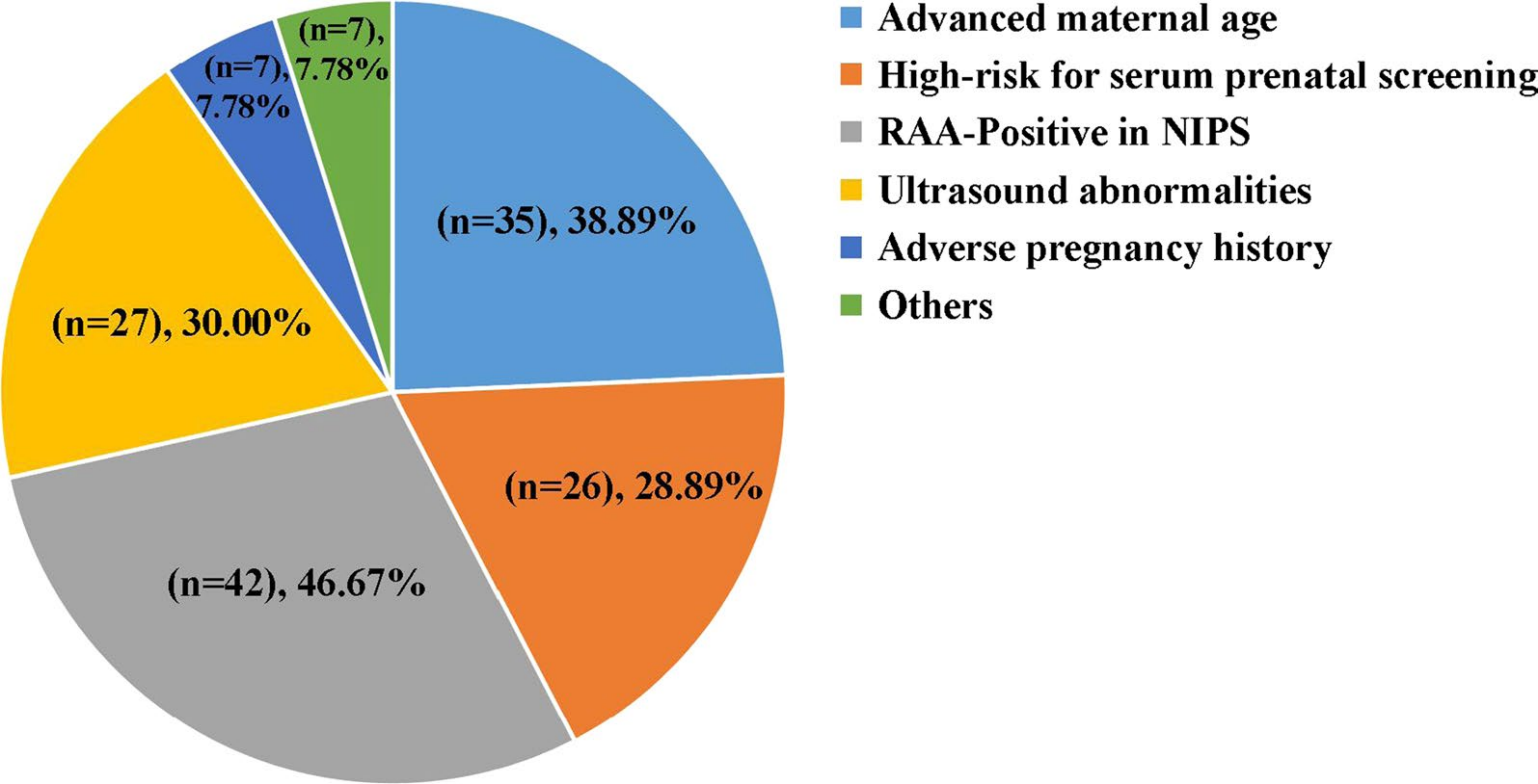
Clinical indications for invasive prenatal diagnosis in RAAs. More than one indication may be observed in each case; thus, the total number of cases in different indications was more than the total RAA cases (*n* = 90). Others include all cases involving high risk of NIPS for trisomies 21, 18, or 13, or parental thalassemia; ultrasound abnormalities include ultrasonic soft markers and structural defects

Of the 90 RAA cases, the four predominant indications for invasive prenatal diagnosis were NIPS RAA-positive (*n* = 42, 46.67%), AMA (n = 35, 38.89%), ultrasound abnormalities (*n* = 27, 30.00%), and high-risk for serum prenatal screening (*n* = 26, 28.89%), as illustrated in Fig. 2. Indications of an adverse pregnancy history and others, including a high risk of NIPS for common trisomies and parental thalassemia, were also observed. Moreover, serial ultrasound monitoring findings showed that 40 RAAs cases (44.44%, 40/90) had ultrasound abnormalities during pregnancy, including soft markers and structural defects (Table 2). Ultrasonic soft markers were observed, including increased nuchal translucency (NT) (*n* = 9), persistent left superior vena cava (PLSVC) (*n* = 6), fetal ventriculomegaly (*n* = 4), and single umbilical artery (SUA) (*n* = 3). Cardiovascular defects (*n* = 14) were the most frequently observed structural abnormalities, followed by musculoskeletal anomalies (*n* = 7) and diaphragmatic hernia (*n* = 2). Additionally, five RAA cases associated with IUGR were found, involving chromosomes 22, 16, 15, and 8 (Table 2).

**Table 2:** Clinical manifestations and outcomes in each RAA case

	Chromosomes involved with RAA														
	16	22	9	2	15	7	3	8	4	12	14	20	5	10	Total
Total	20	16	15	8	8	7	3	3	2	2	2	2	1	1	90
Advanced maternal age	3	8	6	5	5	3		1	1		1	1	1		35
High-risk for serum prenatal screening	9	6	3	2		1			2	1		1		1	26
RAA-Positive in NIPS	10	9	7	2	5	4	2	1			1			1	42
Ultrasound abnormalities	8	8	9	4	1	1	1	1	2	1	1	2		1	40
Soft markers															
Increased NT (≥ 3.0 mm)		4	1	1			1					1		1	9
Persistent left superior vena cava		1	3	1	1										6
Mild fetal ventriculomegaly (between 10 and 15 mm)	1	1	1			1									4
Single umbilical artery		1	1	1											3
Short fetal femur length		1	1			1									3
Echogenic fetal bowel			1	1											2
Echogenic intracardiac focus			1												1
Enlarged cisterna magna		1													1
Renal echo enhancement			1												1
Absent gallbladder			1												1
Structural defects															
Cardiovascular	4	1	3	1		1		1	1	1	1				14
Musculoskeletal	4		1	1		1									7
Diaphragmatic hernia			1	1											2
Urogenital			1												1
Respiratory	1														1
Intrauterine growth retardation	1	2			1			1							5
Pregnancy outcomes															
TOP	16	8	14	5	6	4	2	2	2		2	1		1	63
Live birth	4	8	1	3	2	3	1	1		2		1	1		27
Premature delivery	1	2		2		1									6
Postnatal anomaly	2	1		1	2	1				1					8

*NT* nuchal translucency, *NIPS* noninvasive prenatal screening, *TOP* termination of pregnancy

Pregnancy outcome data were available for all 90 RAAs. There were 63 (70%, 63/90) induced abortions and 27 (30%, 27/90) live births (Table 2). In the live birth group, cases of premature deliveries (*n* = 6) and postnatal anomalies (*n* = 8) were recorded. For the seven complete trisomies, six cases ended in a late termination, of which one case of non-mosaic trisomy 9 was detected from AF that progressed to the second trimester. The remaining case, a non-mosaic trisomy 22 detected from CVS, resulted in a live birth without apparent abnormalities. The clinical findings, molecular cytogenetic results, and outcomes of 90 fetal RAA cases were presented in more detail (Additional file 1).

### Chromosomal analysis of RAAs

To compare the results between different testing methods and specimen types, the RAA cases verified by traditional cytogenetic methods (karyotyping or FISH) were further analyzed. Subsequently, 33 prenatal RAA cases with available cytogenetic results were included. TFMs were recorded in 20 RAA cases based on the consistency between karyotyping and CMA results from AF or CB (Table 3). For case 67, an interphase FISH for further identification confirmed a true mosaic trisomy 16 with a mosaic ratio of 15%, but a discrepancy between karyotyping and CMA results was found. Notably, a discordant karyotyping analysis between AF and CB was also noted (case 71), and FISH performed on AF and CB samples revealed a true mosaic trisomy 16 finally. Besides, the discordant chromosomal results were also summarized (Table 4). There were four cases with abnormal CVS results, three of which were confirmed to be CPMs with normal AF results (cases 5, 15, and 65), but one case had an approximately 8.46 Mb deletion on 21q21.1-q21.3 detected from AF (case 82). Moreover, another seven rare mosaic trisomies were detected from AF via CMA, while the AF karyotyping results were normal (cases 14, 19, 47, 48, 56, 76, and 77). Two RAA cases involving chromosomes 9 and 22 did not proceed to amniocentesis (cases 39 and 81).

**Table 3:** True fetal mosaicisms confirmed based on the consistency between karyotyping and CMA results from AF or CB

Case	MA	GA	Clinical manifestation	NIPS result	CMA result	Karyotyping/FISH result	Outcome
1	39	19	AMA; echogenic fetal bowel	Increased chr2	AF: arr(2) × 3[0.1]	AF: mos47,XN, + 2[8]/46,XN[19]	Premature delivery
4	29	23	High risk of MSS; coarctation of aorta; SUA; PLSVC; rocker-bottom foot	Increased chr2	AF: arr(2) × 3[0.22]	AF: mos 47,XN, + 2[2]/46,XN[33]; nuc ish(D2Z1X3,BCRX2) [6/100]	TOP
6	42	18	AMA; high risk of MSS	–	AF: arr(2) × 3[0.45]	AF: mos 47,XN, + 2[1]/46,XN[24]	TOP
17	31	16	–	Increased chr7	AF: arr(7) × 3[0.1]	AF: mos 47,XN, + 7[2]/46,XN[16]	TOP
20	28	23	Mild fetal ventriculomegaly; short femur length; atrial septal aneurysm; foot varus	–	AF: arr(7) × 3[0.3]	AF: mos 47,XN, + 7[4]/46,XN[18]	TOP
25	42	23	AMA; renal echo enhancement; echogenic intracardiac focus; left ureteral dilatation	–	AF: arr(9) × 3[0.4]	AF: 47,XN, + 9[6]/46,XN[18]; CB: mos 47,XN, + 9[2]/46,XN[98]	Live born
26	37	23	AMA; short femur length; SUA; diaphragmatic hernia	–	AF: arr(9) × 3[0.36]	AF: mos47,XN, + 9[7]/46,XN[15]	TOP
28	30	20	High risk of MSS; absent gallbladder	High risk of NIPS for chromosome 21	AF: arr(9) × 3[0.24]	AF: mos 47,XN, + 9[5]/46,XN[28]	TOP
29	28	19	PLSVC	Increased chr9	AF: arr(9) × 3[0.15]	AF: mos 47,XN, + 9[10]/46,XN[21]	TOP
31	26	17	–	Increased chr9	AF: arr(9) × 3[0.45]	AF: mos 47,XN, + 9[12]/46,XN[8]	TOP
33	40	32	AMA; mild fetal ventriculomegaly; PLSVC	–	CB: arr(9) × 3[0.39]	CB: mos 47,XN, + 9[3]/46,XN[97]	TOP
36	31	15	-	Increased chr9	AF: arr(9) × 3[0.5]	AF: mos 47,XN, + 9[3]/46,XN[27]	TOP
38	40	13	AMA; thicken NT	–	CVS: arr(9) × 3 AF: arr(9) × 2–3[0.83]	CVS: mos 47,XY, + 9[3]/46,XX[35] AF: mos 47,XN, + 9[10]/46,XN[20]	TOP
40	28	18	Adverse pregnancy history; high risk of MSS; thicken NT	Increased chr10	AF: arr(10) × 3[0.59]	AF: mos 47,XN, + 10[4]/46,XN[32]	TOP
42	20	23	Coarctation of aorta; hypoplastic aortic arch	–	AF: arr(12) × 3[0.39]	AF: mos 47,XY, + 12[1]/46,XY[31]	Liveborn
57	35	23	AMA; high risk of MSS; ventricular septal defect; total abnormal pulmonary venous drainage	Increased chr16	AF: arr(16) × 3[0.1]	AF: mos 47,XN, + 16[4]/46,XN[17]	Postnatal death
67	29	16	–	Increased chr16	AF: arr(16) × 3[0.15]	AF: Normal (Karyotyping); nuc ish (D16Z3 × 3,BCR × 2)[15/100]	TOP
68	33	18	High risk of MSS	Increased chr16	AF: arr(16) × 3[0.2]	AF: mos 47,XN, + 16[1]/46,XN[25]; nuc ish (D16Z3 × 3,BCR × 2)[37/100]	TOP
71	32	20	High risk of MSS; IUGR; butterfly vertebra	Increased chr16	AF: arr(16) × 3[0.34]	AF: mos47,XN, + 16[1]/46,XN[19]; nuc ish (D16Z3 × 3, BCR × 2) [15] / (D16Z3 × 2, BCR × 2) [185]; CB: Normal (Karyotyping); nuc ish (D16Z3 × 3, BCR × 2) [7] /(D16Z3 × 2, BCR × 2) [193]	TOP
75	36	20	AMA	Increased chr22	AF: arr(22) × 3[0.15]	AF: mos47,XN, + 22[2]/46,XN[18]	Premature delivery

*chr* chromosome, *MA* maternal age (years old), *GA* gestational age (weeks), *CVS* chorionic villus sampling, *AF* amniotic fluid, *CB* cord blood, *NIPS* noninvasive prenatal screening, *CMA* chromosomal microarray analysis, *FISH* fluorescence in situ hybridization, *AMA* advanced maternal age, *MSS* maternal serum screening, *NT* nuchal translucency, *SUA* single umbilical artery, *PLSVC* persistent left superior vena cava, *IUGR* intrauterine growth retardation, *TOP* termination of pregnancy

**Table 4:** Discordant chromosomal results in fetuses with RAAs

Case	MA	GA	Clinical manifestation	NIPS result	CMA result	Karyotyping/FISH result	Outcome
5	32	14	Adverse pregnancy history	–	CVS: arr(2) × 3[0.23] AF: Normal (CMA)	AF: Normal (Karyotyping)	Normal, Liveborn
14	40	12	AMA; adverse pregnancy history	–	AF: arr(5) × 3[0.6]	CVS: Normal (Karyotyping) AF: Normal (Karyotyping)	Normal, Liveborn
15	32	13	High risk of MSS	–	CVS: arr(7) × 3[0.15] AF: Normal (CMA)	CVS: Normal (Karyotyping) AF: Normal (Karyotyping)	Premature delivery
19	38	19	AMA	Increased chr7	AF: arr(7) × 3[0.1]	AF: Normal (Karyotyping)	TOP
47	28	21	AMA; adverse pregnancy history	–	AF: arr(15) × 3[0.44]	AF: Normal (Karyotyping)	TOP
48	34	20	–	Increased chr15	AF: arr(15) × 3[0.1]	AF: Normal (Karyotyping)	TOP
56	29	19	High risk of MSS;	Increased chr16	AF: arr(16) × 3[0.1]	AF: Normal (Karyotyping)	TOP
65	36	12	AMA; adverse pregnancy history; parental thalassemia	–	CVS:arr(16) × 3[0.15] AF: Normal (CMA)	AF: Normal (Karyotyping) AF: Normal (FISH)	Normal, Liveborn
76	37	16	IUGR	Increased chr22	AF: arr(22) × 3[0.1]	AF: Normal (Karyotyping)	TOP
77	25	16	AMA; high risk of MSS	Increased chr22	AF: arr(22) × 3[0.25]	AF: Normal (Karyotyping)	Normal, Liveborn
82	31	14	Thicken NT	–	CVS: arr(22) × 3[0.4] AF: arr[GRCh37] 21q21.1q21.3(19314275_27775306)x1dn	AF: 45,XN,psu dic(22;21) (p12;q22)del(21)(q21q21)dn	Normal, Liveborn

*chr* chromosome, *MA* maternal age (years old), *GA* gestational age (weeks), *CVS* chorionic villus sampling, *AF* amniotic fluid, *CMA* chromosomal microarray analysis, *FISH* fluorescence in situ hybridization, *AMA* advanced maternal age, *MSS* maternal serum screening, *NT* nuchal translucency, *SUA* single umbilical artery, *IUGR* intrauterine growth retardation, *TOP* termination of pregnancy, *dn* de novo

## Discussion

Recent large-scale NIPS studies have focused on the common trisomies 21, 18, and 13, but limited information is available on prenatal RAAs. Moreover, the variability and unpredictability of phenotypes are the most crucial challenges in counseling prenatal mosaicism. In this study, we analyzed a large sample of 48,904 cases undergoing prenatal diagnosis using CMA, and a total of 90 prenatal RAAs were found. Furthermore, the phenotypes and clinical outcomes of RAAs were elucidated, providing a scientific basis for genetic counseling and pregnancy management.

In general, fetuses with common trisomies 21, 18, and 13 can have a live birth, but those with RAAs usually result in an early abortion [1]. Mosaic trisomy is often detectable when amniocentesis is performed in the second trimester and may be mechanistically related to chromosome non-disjunction, anaphase lag, endoreplication, or UPD [19, 20]. Of the 90 RAAs identified in our study, 83 were mosaic and 7 non-mosaic trisomies, thus confirming that RAAs diagnosed during pregnancy are usually mosaic. As the outcome for most RAA cases was the termination of pregnancy in our study, the subsequent ultrasound findings may be limited. Moreover, the phenotypes and outcomes of mosaicisms are highly complex and variable, which may be associated with the level and tissue distribution of abnormal cell lines [14, 21]. Hence, there is an urgent need to provide reasonable and appropriate genetic counseling for patients.

In this study, four prominent indications for prenatal diagnosis were NIPS RAA-positive, AMA, ultrasound abnormalities, and high-risk for serum prenatal screening. As we know, the associations between AMA and aneuploidies have been reported, especially in common trisomies 21, 18, and 13 [22]. Ultrasound abnormalities, including soft markers and structural abnormalities, are strong indicators of chromosomal abnormalities [23]. Currently, NIPS is recommended as a screening method for common trisomies 21, 18, and 13, and some studies have demonstrated that NIPS plays an important role in RAA verification [24, 25]. In our series, 46.67% (42/90) of cases had NIPS RAA-positive results, the most common indication for CMA, confirming that NIPS was sensitive and effective for the screening of prenatal RAAs. In a recent study, the rate of detectable RAAs was found to vary between 0.12% and 1.03%, which was summarized from ten genome-wide studies based on cfDNA testing [26]. The overall frequency of RAAs observed through CMA in our study was 0.18% (90/48,904), which was lower than that reported in previous NIPS studies [5–8, 25, 27]. As the NIPS result from the maternal plasma cfDNA closely resembles the cytogenetic result from CVS culture [28], the lower detectable rate of RAAs in our study may be attributed to the false positives of NIPS and the possibility of placental mosaicism. RAA detection through CMA can decrease the risk of false positives caused by placental trophoblasts, allowing a reasonably confident confirmation of CPM or TFM.

Based on the available follow-up cytogenetic analysis, TFMs were confirmed in 20 RAA cases based on the consistency between karyotyping and CMA results from AF or CB. In addition, we found discrepant results across various techniques or specimen types in this study. First, some RAA cases had a discordant chromosomal result between the CVS and AF, with a CPM usually being established. A common finding is that a prenatal RAA detected from CVS is a CPM in approximately 97% of cases [29]. Compared with AF, CVS was prone to a false-positive result caused by CPM. Second, a discordant cytogenetic result was also observed between karyotyping of AF and CB. As these specimen types represent different fetal tissues, the discrepancies may be related to the different distribution of trisomy cells in tissues [14]. Third, karyotyping analysis did not coincide with the CMA results. A few cases of low-level mosaicism were revealed by CMA, with an RAA-positive result shown in NIPS. However, the karyotypes of cultured amniocytes were normal, suggesting that a false negative for karyotyping was caused by the disappearance of abnormal trisomy cell lines in conventional amnio-cell culture. Accordingly, it is essential to apply a combination of more comprehensive and accurate techniques, such as CMA and FISH, to uncultured AF or CB for determining prenatal mosaicisms [13, 14].

Furthermore, the most common RAAs detected in our study involved chromosomes 16, 22, and 9, which had previously been reported to be major RAA findings in some NIPS studies [6, 7, 30]. According to a previous study [4], pregnancies involving rare mosaicisms for chromosomes 9, 16, and 22 had a very high risk of abnormal outcomes. Nevertheless, owing to the uncertainty of abnormal cell line levels and distribution, the prenatal phenotypes and outcomes are unpredictable. Therefore, we focused on analyzing the genotype–phenotype correlations of the RAAs detected. Ultrasound monitoring findings showed that 40 (44.44%, 40/90) fetuses had ultrasound abnormalities in our RAA cases. Cardiovascular defects were the most frequently observed structural abnormalities, followed by musculoskeletal anomalies. In addition, the major soft marker abnormalities found were increased NT and PLSVC. As for the RAAs involving chromosome 16, some cases showed abnormal findings such as cardiovascular defects, musculoskeletal malformations, or IUGR, which have been described in the related literature [31, 32]. The few live births in our series also had postnatal abnormalities of congenital heart disease and finger deformities. Since the pregnancies were terminated in most cases, the phenotype spectrum collected was insufficient to correlate the specific malformations with the RAAs. Thus, the importance of comprehensive counseling and subsequent ultrasound monitoring cannot be overemphasized.

For the RAAs involving chromosome 22, the second-most commonly detected in our cohort, four fetuses were found to have an increased NT (≥ 3.0 mm). Moreover, single cases with soft marker abnormalities including PLSVC, mild fetal ventriculomegaly (between 10 and 15 mm), SUA, and enlarged cisterna magna were also included. These soft markers are usually used as prenatal screening indications for common trisomies, and some authors have reported that nuchal thickening was observed in trisomy 22 fetuses in the first-trimester screening [33, 34]. Intriguingly, six pregnancies involving low-level mosaic trisomy 22 proceeded to a successful birth, in which one showed abnormalities including postnatal developmental retardation, hydrocele testis, and ventricular septal defect. Mosaic trisomy 22 fetuses with normal outcomes have also been reported [35]. IUGR, facial cleft, cardiac anomalies, and oligohydramnios were the prominent features previously reported in mosaic trisomy 22 fetuses [33, 34, 36]. However, no craniofacial abnormalities or oligohydramnios were observed in our cases, demonstrating that these anomalies may not occur in all prenatal cases. The relationship between the specific phenotypes and the chromosomes involved still needs to be verified in further studies.

Mosaic trisomy 9 is often associated with multiple organ malformations, such as facial feature, cardiovascular, skeletal, and genitourinary abnormalities [37]. In our series, one fetus presented with multiple malformations involving facial abnormalities, punctate epiphyseal dysplasia, and myocardial thickening. A few fetuses had abnormal heart structures involving the right aortic arch and persistent truncus arteriosus. Notably, more soft marker abnormalities in RAAs involved chromosome 9 were involved, including increased NT, PLSVC, mild fetal ventriculomegaly, SUA, echogenic intracardiac focus, and renal echo enhancement. Moreover, a single case of complete trisomy 9 that proceeded to the second trimester was detected in the AF, with an abnormal ultrasound finding of persistent truncus arteriosus resulting in termination, suggesting that trisomy 9 sometimes occurs even in the second trimester in a non-mosaic form [38]. There was only one live birth involving mosaic trisomy 9, but a follow-up was refused.

In addition, two live births with low-level trisomy 15 mosaicism were recorded. One case showed postnatal molecular genetic evidence of Prader–Willi syndrome, which is often due to UPD caused by meiotic non-disjunction following trisomic zygote rescue, especially with maternal non-disjunction of chromosome 15 [39]. The other case with mild motor retardation and IUGR was recorded, which had been described in some case reports [40]. RAAs involving chromosome 7 are also frequently detected in some NIPS studies [6, 7, 30], but were not frequently observed in our cohort. Only one fetus showed ultrasonic anomalies of lateral ventricle dilation, short fetal femurs, atrial septal aneurysm, and foot varus. Another case utilized follow-up CMA of the parents and excluded UPD 7, and the fetus did not show apparent ultrasound abnormalities. The pregnancy was finally terminated. As chromosomes 15 and 7 are known to be associated with imprinting or parent-of-origin effects, a child–parent trio analysis through CMA is often required in clinical practice [41]. However, the end of pregnancy in most of our RAA cases, especially when ultrasound abnormalities occurred, resulted in the unavailability of UPD analysis. Notably, a rare case with 25% mosaicism for trisomy 3 presented with thickened NT (4.2 mm) and a tethered spinal cord, without other structural abnormalities. An uncommon RAA case of mosaic trisomy 12 presented with cardiac anomalies, including coarctation of the aorta and hypoplastic aortic arch, which had a favorable outcome after undergoing cardiac surgery. Limited cases of these infrequent RAAs are more prone to biases, and the lack of consistency among phenotypes makes it difficult to evaluate the hallmark features of RAAs.

This pilot study had several limitations. Our data were obtained retrospectively from the samples that had undergone CMA in the Guangdong Women and Children Hospital, and follow-up confirmatory detection was limited. Moreover, induced abortion was observed in most of our cases, which may have led to incomplete clinical manifestations on subsequent sonographic findings. Therefore, genetic counselors should combine serial ultrasound monitoring, multiple diagnostic methods, and complete follow-up for the prenatal diagnosis of RAAs.

## Conclusions

The overall frequencies, clinical manifestations, and pregnancy outcomes of prenatal RAAs were analyzed in this study, which can provide informative and valuable knowledge for genetic counseling on RAAs. Moreover, the phenotypes and clinical outcomes were heterogeneous in RAA cases. Thus, a cautious and comprehensive strategy should be included in future counseling for prenatal RAAs.

## Methods

### Patients and data collection

All patients who underwent invasive prenatal diagnosis via CMA in the Guangdong Women and Children Hospital between April 2016 and December 2020 were included in this study. This tertiary referral center provided the referral services for the primary and neighboring hospitals to recruit a sufficient number of patients. Clinical samples (chorionic villi, amniotic fluid, and cord blood) were obtained by ultrasound-guided transabdominal chorionic villus sampling (CVS), amniocentesis, or cordocentesis. CPM was identified when an abnormal result was found in CVS, and further analysis of amniocytes was normal. True fetal mosaicism (TFM) was identified based on the results of amniotic fluid (AF) or cord blood (CB) cytogenetic studies. Each participant signed a written informed consent form after accepting detailed pretest genetic counseling. This study was approved by the Ethics Committee of Guangdong Women and Children Hospital (number 202201012).

Clinical indications, including advanced maternal age (AMA), high risk for serum prenatal screening, RAA-positive in NIPS, ultrasound abnormalities, adverse pregnancy history, high risk of NIPS for trisomies 21, 18, and 13, and parental thalassemia, were collected in this study.

### CMA

Fetal uncultured genomic DNA was extracted using a DNA extraction kit (QIAamp DNA Mini Kit, QIAGEN, Germany). CMA was performed using a whole-genome CytoScan 750K array (Thermo Fisher Scientific, USA), as recommended by the manufacturer. The raw data were analyzed with the Chromosome Analysis Suite 4.0 (Thermo Fisher Scientific, USA) based on the genome version GRCh37/hg19. Mosaic trisomy was reported when the median calibrated log2 ratio of a chromosome was between 2.10 (10%) and 2.90 (90%). When a low-level mosaic trisomy (10% to 30%) was found by CMA, karyotyping and an interphase FISH were recommended to further verify, and the NIPS result and clinical manifestation should be taken into account if available.

### G-banding and FISH

Cells were cultured and prepared for G-banding and FISH following standard protocols. Karyotypes were described based on the criteria of the International System for Human Cytogenetic Nomenclature (ISCN 2020) [42]. When a suspected low-level mosaicism was observed, an interphase FISH of uncultured amniocyte cells was recommended to be performed.

### Statistical analysis

Statistical analysis was performed using SPSS 19.0 (Chicago, USA). Categorical variables were presented as percentages, and quantitative variables were presented as medians (interquartile range). Chi-square tests were used for categorical variables, and quantitative variables were analyzed using the Mann–Whitney *U* test. Statistical significance was defined as a two-sided *p* value < 0.05.

## Supplementary Information

**Additional file1: Table S1 **Clinical findings, molecular cytogenetic results, and outcomes of 90 fetal RAA cases.

## Data Availability

The datasets used and/or analyzed during the current study are available from the corresponding author upon reasonable request.

## Abbreviations

RAAs: Rare autosomal aneuploidies
CPM: Confined placental mosaicism
IUGR: Intrauterine growth retardation
UPD: Uniparental disomy
NIPS: Noninvasive prenatal screening
cfDNA: Cell-free DNA
FISH: Fluorescence in situ hybridization
CMA: Chromosomal microarray analysis
CVS: Chorionic villus sampling
TFM: True fetal mosaicism
AF: Amniotic fluid
CB: Cord blood
AMA: Advanced maternal age
NT: Nuchal translucency
PLSVC: Persistent left superior vena cava
SUA: Single umbilical artery
